# Onions

**Published:** 1896-06

**Authors:** 


### Onions.

Onions are almost the best nervine known. No medicine is so useful in cases of nervous prostration, and there is nothing else that will so quickly relieve and tone up a worn-out system. Onions are useful in all cases of coughs, colds and influenza; in consumption, insomnia, hydrophobia, scurvy, gravel, kidney and liver complaints. Eaten every other day they soon have a clearing and whitening effect upon the complexion.—*Exchange.*

